# Is acting on delusions autonomous?

**DOI:** 10.1186/1747-5341-8-14

**Published:** 2013-10-14

**Authors:** Jann E Schlimme

**Affiliations:** 1 Psychiatric University Hospital of Charité at St. Hedwig Hospital, Charité Universitätsmedizin, Große Hamburger Straße 5-11, Berlin 10115, Germany

**Keywords:** Phenomenology, Delusional convictions, Delusional habituality, Self-determination, Autonomy, Goods of agency, Double-orientation to reality

## Abstract

In this paper the question of autonomy in delusional disorders is investigated using a phenomenological approach. I refer to the distinction between freedom of intentional action, and freedom of the will, and develop phenomenological descriptions of lived autonomy, taking into account the distinction between a pre-reflective and a reflective type. Drawing on a case report, I deliver finely-grained phenomenological descriptions of lived autonomy and experienced self-determination when acting on delusions. This analysis seeks to demonstrate that a person with delusions can be described as responsible for her behaviour on a ‘framed’ level (level of freedom of intentional action), even though she is not autonomous on a higher (‘framing’) level (level of freedom of the will), if, and only if, the goods of agency for herself and others are respected. In these cases the person with delusions is very nearly comparable to people in love, who are also not free to choose their convictions, and who could also be rightly held responsible for the behaviour flowing from their convictions.

## Introduction

Persons with delusions often claim to be autonomous if they are behaving in accordance with their delusional convictions. Can this claim be justified? In order to address this question I will develop fine-grained descriptions of experiences of autonomy, or of feeling free and self-determined, if acting on delusions from the first-person-perspective. However, investigating the experiential structure of being autonomous from the first-person-perspective faces a fundamental problem: employing methods that focus on the experiential structure from the first-person-perspective, like phenomenological methods, offer descriptions of the experiential structure, but not moral justifications. The phenomenological method, as applied in this paper, might therefore not appear well suited for this question. Nonetheless, it would be absurd if experiences of autonomy would not differ somehow from those experiences in which a person is, in spite of her own experience of feeling free and self-determined, not autonomous. Possible features of such a self-deception in delusions could be the impossibility of falsifying one’s convictions during ongoing delusions and the highly private character of delusional convictions not shared with one’s community. Hence the question remains: Is a person with delusions simply and generally deceived regarding a given experience of autonomy, if acting on delusions?

In order to proceed, I first present a short phenomenological description of delusional convictions, mainly drawing on the German tradition of phenomenological psychopathology (Phenomenological psychopathology of delusional convictions section). In a subsequent (The experiential structure of lived autonomy section) I provide a preliminary phenomenological description of lived autonomy, drawing on recently published work, and will then discuss lived autonomy in delusions more closely (Delusion-related alterations of lived autonomy section). In this Introduction section offer a detailed description of delusion-related alterations of lived autonomy, whether on the level of freedom of intentional action or of freedom of the will. Basically, I argue that freedom on the level of intentional action can be sufficient for profound autonomy, if the goods of agency are respected; and I attempt to demonstrate that persons with delusions are, in this respect, partially comparable – although with notable distinctions – to people in love. Lastly, in Limitations of lived autonomy in delusions section, I discuss this argument with respect to clinical aspects.

### Phenomenological psychopathology of delusional convictions

Delusional convictions can be understood as highly private, explicitly known and incorrigible convictions which have gained the status of a ‘justified belief’, although being unjustifiable (and non-falsifiable) in discourse. According to the German tradition of phenomenological psychopathology two criteria can be named for delusional convictions: a) the extraordinary degree of conviction concerning particular ideas; and b) the outright rejection of alternative explanations, the so called *Unkorrigierbarkeit* (incorrigibility) of these convictions (ideas). The impossibility of the delusional content, which is often the most prominent feature in clinical settings, is only an accessory criterion for the psychopathological finding of delusional convictions. It merely indicates the private character of these convictions (see p. 216 ff; p. 157 f.) [1-3]. These psychopathological criteria mirror the well-known fact that a deluded person’s feelings, valuings, thoughts and behaviour are pre-reflectively prescribed by her delusional convictions. We can therefore summarize that there are two characteristics distinguishing delusional convictions from other and (voluntarily) self-imposed commitments:

a) The person with delusions is pre-reflectively altered according to these convictions in the sense that her critical abilities are in line with her delusional convictions. In short: The deluded person cannot be self-critical with respect to her delusional convictions [4]. This is a classical insight in psychopathology (see already Sandberg [5]; Jaspers [6] p. 46: “Die Kritik […] stellt sich in den Dienst des Wahns”). Of course, one’s pre-reflective structure corresponds (more or less) one’s basic convictions. Importantly, however, the person with delusions need not try to convince herself of her delusional convictions; she always already is convinced. This holds true even if she tests the validity of her delusional convictions in the course of their development, because they appear just too bizarre from her point of view.

b) The deluded person is (“*per definitionem”*) ineffective and helpless with respect to altering these convictions. This missing effectivity is the basic impairment in persons with delusions. It is also an impairment of her freedom of the will. In other words: a person with delusions is pre-reflectively altered in such a way that she is unable to retrospectively find out for herself that she was and still is deceived. This is the highest form of self-deception. In other words: as long as the delusions are maintained, such self-deception is undetectable. The person with delusions is not able, despite all changes in her life situation or her attitude, to reframe these convictions as interpretations or justified beliefs (else she would not hold these delusional convictions any more). Nonetheless, she might be able, as I will argue in this paper with reference to a case report, to momentarily suspend her delusional experiences adopting a more common-sensical attitude without truly criticizing her delusional convictions (called a ‘double-orientation to reality’, see Jaspers, p. 101; 4; [6-9]).

### The experiential structure of lived autonomy

In this section I will argue for a distinction between two different types of lived autonomy: a pre-reflective and a reflective type of lived autonomy. This distinction is a very broad distinction. Nonetheless, it allows for profound phenomenological descriptions of symptom-related alterations of lived autonomy, as I demonstrated in recent publications [10,11]. What is meant with these two types of lived autonomy?

Autonomy requires us to behave responsibly and to be able to provide appropriate reasons for our behaviour. Usually, we would expect such a moral agent to be able to display the following abilities: to develop intentions for his behaviour, to show a certain effectivity of his behaviour with respect to his intentions, to judge his behaviour independently from his intentions, and to provide reasons for his behaviour. Accordingly, an agent displaying these abilities in a given situation should experience himself as autonomous. For this experience of autonomy, deliberation is required in order to recognise and effectively perform those actions that are in one’s best interest, including one’s moral interests p. 46 [12]. I would like to call this the “reflective type of lived autonomy, which can be experienced if delivering a well-founded (moral) justification for one’s behaviour or one’s behavioural intentions, goals or values, whether one realises them in one’s behaviour or not (‘self-effectivity’) or whether they are in accordance with one’s deepest or long-term interests or not (‘authenticity’). This type involves, of course, freedom of the will, but it may also involve freedom of intentional action (authenticity, effective and responsible self-realisation)” [11].

This reflective type of lived autonomy does not capture our usual experience of being, or feeling, free. On the contrary, we usually experience ourselves as ‘free’ in those situations in which we are not explicitly self-conscious. Nonetheless, in these experiences we, as agents interested in autonomous behaviour, usually pre-reflectively anticipate that we would be able to explicitly evaluate our accustomed behaviour as morally justified (i.e., from some future point of view). Of course, this anticipation does not imply that we would actually judge our behaviour as morally justified if explicitly evaluating it. Nonetheless, if we follow *virtuous* customs, habits or traditions in our behaviour, we might indeed behave in a morally justified manner without explicit deliberation (Drummond [12,13]; see, also, Aristotle [14] 1103a-b). I would therefore like to define the pre-reflective type of lived autonomy as a type of autonomy, “which can be experienced through customary behaviour or in those actions in which we are focused on the things at hand. This type may, of course, involve freedom of the will, but it corresponds at least a freedom of intentional action” [11].

From a phenomenological point of view, both types of lived autonomy are prescribed by our habituality, that is: our habitualised perceptions and value-apprehensions, our styles of thinking and behaving, our values and life-goals (‘projects’) and our current emotional states and interests, as has especially been pointed out by John J. Drummond [12,13,15,16]. Habituality is a phenomenological term that describes the passive and continuous disclosure of one’s life-world (*Lebenswelt*) and oneself in the same constitutive manner. If this habituality of a person is informed by virtues, she can indeed behave in a morally justifiable manner, rendering this person autonomous in the sense of an implied freedom of the will. Insofar, our phenomenological description of lived autonomy obviously coincides with the fact that the majority of lived autonomy is given in habitualised behaviour. This may explain why models of virtuous ethics seem to be closely related to moral agency [13,15]. Besides, living autonomously can, in itself, become familiar and habitualised, entailing pre-reflective intentions of anticipation with respect to the (moral) justification of one’s customary behaviour.

Nonetheless, a person’s autonomy that would merely rely on tradition and habituality could be arbitrary. In order to behave autonomously in the situation in which one is called upon to act, it is, not surprisingly, often required to explicitly take one’s perceived action-properties, pre-reflective valuations or willing acts into account, and, hence, reflectively identify and evaluate the goals, interests and values inherent in one’s pre-reflective mental operations. This can entail experiences of lived autonomy corresponding an explicitly chosen, well-defended and justified behaviour (reflective type of lived autonomy). Experiences of this reflective type of lived autonomy may incrementally alter one’s habituality and might therefore enable a more virtuous or authentic manner of one’s pre-reflective behaviour in the long run (pre-reflective type of lived autonomy). Nonetheless, this autonomy-spiral does not render repeated reflection unnecessary. On the contrary, since an autonomous and responsible manner of life-conduct can never be taken for granted, it is constantly required to guard one’s own and other’s right to value, judge, decide and behave for oneself (so called ‘goods of agency’; Drummond). Drummond captures this goal of autonomous and responsible behaviour in the concept of *authenticity*, arguing that authentic behaviour means the successful realisation of those values the person in question values most (with respect to her situation and life-conduct). “The authenticity of this kind of life is responsible self-realization, taking responsibility for one’s convictions and for disclosing the evidence that warrants those convictions (for oneself or others J.S.)” [16] p. 423.

### Habituality and familiar workspace

Habituality is undergirded, as Husserl argued in his ‘genetic phenomenology’, by pre-reflective intentions of anticipation and their ongoing fulfilment (“*Erwartungsintentionen*”, Husserl [17], §21-26). For example, the shape and style of the objects in our situation as well as of many circumstances of our situation are prescribed precisely by our intentions of anticipation on the perceptive (‘doxic’) level. Furthermore, we perceive objects or circumstances as usable or manageable in this or that manner. They display specific action-properties, which we perceive immediately as an invitation or option to handle them specifically (called *Zuhandenheit*, Heidegger [18], p. 68 ff.; see also recently [19]). To perceive a cup (as a cup) implies not only the ‘perception’ of its bottom and backside, even though we cannot see it at this moment, but also its usefulness for holding liquids (e.g., hot tea). In this habitualised ‘production’ of *Zeug*, (i.e., a “thing”; and what it takes to be such a thing) pre-reflective anticipations of our capabilities guide our focus of consciousness towards those goals that are achievable (and often, though not always or necessarily, valuable) for us in the given situation, typically according to our momentary project we are engaged with. Both the perceived action-properties and the anticipated behavioural effectivity (action-abilities) as well as their experienced fulfilment usually remain pre-reflective in accustomed behaviour. They undergird possible elaborate and explicit expectations with respect to the desired effects, unwanted side effects and the effectivity of our behaviour. Thus we, as embodied selves, habitually disclose our own life-world to ourselves in the manner of a *familiar workspace* and are thus at least usually embedded in at least partially familiar situations (Schlimme [10,11]; ‘experiential workspace’ Talero [20]; similarly, Merleau-Ponty [21], pp. 164 ff.).

Disclosing the perceived action-properties of the objects and circumstances of our life-world according to our habitualised intentions of anticipation describes only one feature of our familiar workspace we inhabit as agents. A second feature is the immediate and *prima facie* valuing (“*Wertnehmungen*”; “value-apprehensions”, valuing without further and explicit value-judgement) of these non-axiological (action-)properties perceived in our life-world. As Husserl noted, valuing acts are not objectifying. Values are not material objects. Of course, pre-reflective value-apprehensions can be objectified secondarily in a reflective act as abstract values. Nonetheless, it is important to recognise that valuations refer to specific properties of perceived objects or actual circumstances: “The value-apprehensions belong to a different dimension. This dimension requires a further dimension as a basis in which objectivity is already fully constituted”. (Husserl [22], XXVIII, p. 262).

From a phenomenological vantage point, we can describe these pre-reflective valuing experiences as being founded on a purely descriptive ‘objective sense’ of an experienced object as the core of this experience. Founded upon this core is a ‘feeling-moment’ in the particular kind of act called (pre-reflective) valuing (“*Wertnehmung*”). As Drummond has stated, the specific moment of this act is “the affective response to the situation with its non-axiological properties” [12], p. 41. Since only some features of this presented (or disclosed) object are addressed in this affective response (we experience objects as bad, brute, elegant, fabulous, fresh, frightening etc.), there is “something like an abstraction at work in evaluation” [13], p. 19. This immediate and pre-reflective abstraction, or particular selection that takes place in the act of valuing, is prescribed by our (pre-reflective) interests, our habituality and momentary moods and by the ‘projects’ we are engaged with [11,12] p. 22 ff. Drummond summarises this idea in a recent paper: “Value-attributes are the correlates of […] the affective response of a subject with a particular experiential history – that is, particular beliefs, emotional states, dispositions, practical interests, and so forth – to the non-axiological properties of an object or situation” [16], p. 416.

The immediate and pre-reflective evaluations of the properties of an object or situation also refers to action-properties, which are disclosed to oneself in one’s actual situation according to one’s habituality and the ‘project’ one is engaged with. These action-properties, which indicate the ‘possibility space’ of the person called upon to act, are immediately and pre-reflectively (so to say, in a ‘second step’) affectively responded to. Thus some action-properties might be valued as suitable and worthwhile and others as impractical and, hence, only providing the context of the behaviour one is about to choose. In other words, goals that are valued as ‘worthy of pursuit’ are already pre-reflectively shaped as the attractive ones according to our habituality and the ‘project’ we are actually engaged with and disclosed as attractive behavioural options in our familiar workspace. This worthiness is, at least sometimes coinciding and fuelled by our pre-reflective anticipation of being able to perform successfully and to achieve intended and worthwhile goals as a result.

These descriptions argue for foundational relations between different levels of our mental lives. The perception of action- or project-properties can be addressed as the basic level. The next level is the level of pre-reflective valuations, which is connected with the doxic level of perception by an affective response to these properties. Based upon these two layers of habitually disclosing a familiar workspace (action-properties and the valuing of these properties) is the conative level of willing acts. Willing acts are directed by goals and goods that are intertwined with the project one is actually engaged in, whether explicitly recognized or not, and respond to perceived and valued action- or project-properties by willing them into existence (*fiat*). Adopting a certain project-related attitude therefore implies to disclose the familiar workspace in a certain, usually corresponding manner. Consequently, one’s willing acts automatically aim at project-relevant goals in their pre-reflective response to adequately valued action-properties. If these intentions of willing certain properties into existence become fulfilled, since these properties can actually be perceived, this “perceptual certainty (generates) the quality of being born out of one’s willing” (Husserl [22], XXVIII, p. 107, see p. 111). Depending on the perceived non-axiological property and its context within the project one is engaged in, this can also entail an experience of (more or less) effectively pursuing one’s goals, whether in pre-reflective valuing or explicit evaluation.

The effectivity of one’s behaviour in order to pursuit and achieve one’s intended goal might not be object of explicit deliberation if things are running smoothly, herewith maintaining and supporting one’s habituality. Nonetheless, this might be different if unwanted or unpleasant effects derive, or one’s behaviour remains ineffective regarding one’s intended goals. All these effects, which might even be mediated by effected others, may imply an explicit moral evaluation of one’s behaviour, goals or even the manner of how one discloses one’s familiar workspace. Drawing on these fine-grained descriptions of lived autonomy, we are now prepared to investigate delusion-related alterations of lived autonomy in a more detailed way in the following section.

### Delusion-related alterations of lived autonomy

A deluded person’s effectivity to pursue and achieve her intended goals is specifically impaired and altered. This is especially true with respect to goals directly deriving from her delusional convictions. For example, you cannot hide effectively from your persecutors. Even if you are able to do so for a shorter period of time, i.e., if you move your household from one apartment to another, your sanctuary will usually not last long. A comparable situation occurs if a deluded person is trying to convince others of her divinity. Sometimes it may even complicate matters if a deluded person uses behavioural strategies in accordance with her delusional convictions.

Mrs. F.,^a^ a 59-year old patient with chronic schizophrenia, repeated psychotic (hallucinatory) experiences and ongoing delusional convictions and very prominent negative symptoms (i.e., abulia, apathy) for over 2 decades, reported that she had had some kind of skin condition at her feet roughly ten years ago and that her grandmother, who had died many years before, talked to her during an ‘apparition’, telling her that this was some kind of “mycosis”. Mrs. F. was delusionally convinced to have met the spirit of her grandmother, having already had frequently apparitions of dead loved ones besides those of completely unknown people. As she described in detail, the bodies of these spirits were “translucent” or even “transparent” and had obviously a different kind of materiality, since “they can walk through walls”. Accordingly Mrs. F. recognized immediately if she faced a spirit or a normal person. She trusted this voice and apparition to be her grandmother’s spirit and treated the skin condition as if it were athlete’s foot, but without any success. Two years later she visited a medical doctor, who provided a totally different diagnosis which proved to be correct, since his treatment quickly improved her condition. She commented on this experience: “The voice led me astray. I cannot trust all voices. It is sometimes very strenuous, to distinguish which voice is trustworthy”. and “I don’t want to take it out on my grandmother, since I don’t know if it was really her”.

Mrs. F. was, at her own discretion, treated during the time when I took field-notes (transcriptions of our therapeutic sessions) without neuroleptic medication, since she suffered extensive extrapyramidal side effects during her first in-patient treatment decades ago. She described this experience as follows: “The thoughts are racing, but the body can do nothing. And the thoughts go like: How shall that work, if I must go to the toilet? And then you are asked: Do you still hear those voices? And when you say ‘Yes’, you get those drops knocked back. Until my guardian angel told me: You have to say ‘No’, otherwise you will never get out here. – It is not easy not to tell the truth”. Mrs. F. did not use any neuroleptics since her release from the hospital, but sometimes used a light sleeping pill for sedation (doxylamine) at a low dose (e.g., 25 mg or less). She frequently went without this medication for days, using it only now and then. Neither her scenic hallucinations, nor any of her other symptoms except repeated sleeplessness were altered in frequency or in intensity by this medication, as her reports demonstrated most clearly.

Curiously, delusional topics are about human features that human beings can never expect to be able to change, but simply have to endure. In other words: in delusions the missing pre-reflective effectivity is ‘perfectly externalized’; ‘perfectly’, because the delusional topic is truly out of range for human beings. In one word: we cannot expect us to change these facts (which are delusional ‘facts’). Mrs. F. was convinced that her basic problem resulted from evil spirits and their influence on her and others. Accordingly, it was paramount to gain at least some influence over these evil spirits. While these evil spirits usually kept away when she was engaged with familiar persons (i.e., her loved ones and family, or professional personnel from our Psychiatric Outpatient Department), she had no direct influence on these highly perplexing and sometimes horrifying apparitions. She received however, at least from her point of view, profound help from her guardian angels: “I believe that good spirits block it so that I don’t receive too much other images or hear things. Otherwise that scares me and I can’t breathe. I sit there and can’t breathe. I think that we all have angels who guard and protect us. They can prevent some things, but some not”. She experienced direct help form her “Great Guardian Angel” (who she identified as the archangel Michael) in the beginning of her psychosis, when her apartment was literally flooded with imagined others: “You don’t have any private life. You can’t take a shower in private. There’s running everyone through your apartment: Grandpa, children, fat and thin ones, people you don’t know. That gets on your nerves, it is just too much. Then I begged my Great Guardian Angel to throw everyone out who didn’t belong to me. It worked! I know that my Guardian Angel will throw anyone out who get’s on my nerves. I feel protected in that regard. – You cannot block the images. They are simply there, but no one ever did anything to me physically. I’m often scared, but, well – you cannot ignore that! You get used to it, get more determined and say: get away! I call them names if they insult me. – During daytime, there’s light and it’s easier. In the night, if you are roused, you are more sensitive. Hideous!” As can be seen, the protection and support by her guardian angels was selective (i.e., no bad spirit ever did anything to her physically, but she was scared and frequently unable to breathe; the guardian angels can prevent some things, but not others) and out of her control (i.e., the angels’ help obviously works better during daytime because of the light, but it is needed more during the night when it is dark).

Nonetheless, the guardian angels offered help and relief from her point of view: “It is a connection of the heart. I feel it immediately, if he (Michael) stands behind me” (which would be called a *leibhaftige Bewusstheit:* awareness of an incarnated other, typically behind oneself and out of sight, from a psychiatric vantage) She even described this position to be the usual one of angels: “You have angels in the fashion of a half-circle behind you”. She reported a scene from her night-shifts when she was (previously) employed in a care facility, roughly four years ago, in which she experienced direct help: “I was on night-shift, it was Sylvester, and I was alone. And he ran through the house in his uniform, just as he laid in bed (note: the resident had died during the daytime and had been robed in his military uniform, J.S.). And he frequently laid down in other peoples’ beds (as when he was still alive, being quite demented, J.S.). […] I was unsure if I just imagined him or not. And I asked my guardian angels, and Michael, to walk in front of me and the others behind me. And it worked. He (the spirit of the man who died that day, J.S.) stood at the staircase and looked up to me, all night. If he was truly a spirit, he must have seen the angels. And if I just imagined him, then I imagined him in adequate fashion”. This report demonstrates besides the gradual realness of her hallucinations, as is frequent in schizophrenia, and the delusional quality of her interpretations, the limit of her angels’ support. They did not chase or guide away the spirit of the dead man, nor did they render him harmless or convince Mrs. F. that he was harmless (for her) in a fundamental sense, or block her imagination (if it was imagined, as she mused). Instead, they protected her outwardly and in a more physical sense, as if they were some kind of supernatural body-guards.

As can be seen, Mrs. F. interpreted her hallucinatory experiences with the traditional concepts of angels, spirits and the like. Being raised in a very traditional Catholic manner, she had believed in the possible reality of angels and evil spirits all her life, and hence immediately valued and retrospectively evaluated her first psychotic experiences as ultimate proof of their presence. Nonetheless, it proved to be a delusional conviction, and not a religious belief, according to modern psychopathological criteria. For example, she did not *believe* in angels, but knew them to be a fact (which is not astonishing taking her experiences into account). Thus, her delusional conviction was judged as a justified belief from her point of view, even though the veridicality of angels can neither be proven nor falsified on rational grounds (see i.e., the classical argument of David Hume on wonders [23], p. 171 ff.). Accordingly, Mrs. F. did not pursue the goal of changing these important ‘facts’, since she already knew that she was unable to change them (being facts from her point of view). Often, this extraordinary conviction of delusional convictions is achieved in the course of the illness and involves different ways of proving, testing and approving the reality of these ‘facts’ in its course. Mrs. F. commented this problem in an off-hand manner: “What is true? What is not true? What is real? You know, when you see your own parents (who died some years before this event), and you talk with them. What shall you believe? […] If you get used to it, then it’s just how it is”. Consequently, Mrs. F. tried to co-exist with these delusional experiences, i.e., visits of friends or loved ones who died long ago, or delusional perceptions. She behaved in accordance with them, and set out goals that were at least not directed against her delusional convictions. This ‘feeling of normality’ did not mean that she got used to these experiences cavalierly. On the contrary, they sometimes still upset her. “I saw a colleague from my night shifts last night (Mrs. F. stopped night shifts years ago). She was a very sweet-natured person who died much too young. This (kind of visit) is not so problematic as i.e., a figure whom you cannot puzzle out. Nonetheless, it’s not really great”. Mrs. F. described in detail how they entered the living-room simultaneously, Elizabeth (pseudonym), her colleague, from the floor through the wall, and she herself coming in from the balcony through the door: “We greeted each other – and that was it”.

The factuality of “this other world”, as she used to call it, did not imply that she could not influence the presence of spirits other than by calling upon her guardian angels. She could, as was already reported, also influence apparitions by being engaged with familiar others. “That is just like rolling thunder in the background (the other world J.S.). Sometimes, if I’m engaged with my grandson, it (the rolling thunder, J.S.) is not even there. But that is only a distraction, it (this other world, J.S.) is always there”. Obviously Mrs. F. could experience a flow-state of mind if being engaged with her grandson, even though she immediately devalued this freedom of action as ‘distraction’ if starting to reflect upon it. From her point of view she was convinced that the evil spirits were never banned, but only absent for a short moment. She reported that highly predictable, repetitive and, with the exception of her young grandson, socially isolated situations were best suited to provoke such ‘experiences of distraction’: “Sometimes I listen to audio books. I do that. That helps, carries me away. I slip out of my situation. I need happy ends. I have listened at least three times to it (a certain audio book with several episodes, J.S.), there is nothing new in it anymore. That works! If something bad happens, I already know that it’s over soon. […] It’s only stories, a different world. That world is defined and fixed, I am acquainted with it, nothing can scare me anymore. That distracts me from this world – It is important to know what is about to come”. However, these strategies were not altogether reliable, especially during the night, as Mrs. F. reported.

Nonetheless, her conceptualization of these psychotic (hallucinatory) experiences as apparitions of spirits or angels was comforting insofar as it provided and offered a comprehensive explanation. Mrs. F. did not provide a precise description of the prodromal and early stages of her psychosis, usually avoiding these questions. Nonetheless, the delusional insight of archangel Michael’s givenness and protection came after a longer period of horrifying and perplexing experiences as described above, most probably involving a stage of *trema,* or delusional mood, as described by Conrad ([24]; see also Mishara [25]). Michael’s help during her first and only admission to a psychiatric ward as well as her religious belief supported the development of a complex delusional system, which helped her to comprehend the otherwise incomprehensible (“… reason tells you that this is impossible”). It was, furthermore, her own activity which led to these delusional insights. Her own ideas helped her to understand the obscure changes going on in her world; it was her mental activity that hammered out the details in her (valuable) explanation for formerly and otherwise incomprehensible changes which took place in her world for over twenty years. It is a classical psychopathological insight that the delusional convictions explaining the otherwise incomprehensible changes in the world are experienced and valued as personally achieved (already Hagen [26]; Sandberg [5]; see also [24]).

There is, furthermore, a missing effectivity in the social dimension. Despite their best efforts, persons with delusions are typically totally unable to convince others that their delusional convictions are true. (A clear exception is *folie à deux* (shared psychosis) which I will not discuss in this paper). Mrs. F. also faced such difficulty in her personal relationships as well as in her professional life. In this social niche she hid her psychotic experiences behind the culturally accepted topic of ‘esoteric phenomena’. In contrast to her own experience, the people calling her could believe her to be a medium or not, to have such apparitions or not. If she had, however, tried to convince her clients to the extent that she was convinced herself, they might call her ‘mad’ or ‘psychotic’. Obviously, interpersonal verification usually evaluates the deluded person’s point of view as ‘incorrect’, ‘non common-sensical’, ‘unusual’, ‘weird’, or even, psychopathologically correctly, as ‘delusional’. In other words, interpersonal support of one’s world- or self-view is seldom available [27]. This often enhances social isolation, an important factor in maintaining delusions (even in *folie à deux*[2,3,27,28].

As the case of Mrs. F. demonstrates, a history of delusional and hallucinatory experiences implies the acquisition of new intentions of anticipation on the perceptual as well as the valuing-level, mirroring her repeated experience of apparitions, whether evil or beneficient, and their reflective consideration. Their initially baffling non-axiological properties (i.e., they have translucent or even transparent bodies that can move through all kinds of materials, but are able to speak and intentionally focus on the scene) are no longer baffling, but accustomed, as well as repeatedly tested and approved. This also entails the perception of certain action-properties that correspond to one’s delusional insights and convictions on the one hand, and one’s experiential history and one’s (behavioural) projects on the other (i.e., you cannot chase them away, but must call for help from your guardian angels instead). Insofar as one’s perceived action-properties and their pre-reflective valuing also mirror one’s former (and possibly accustomed) delusional experiences and one’s actions, behaviours or projects are answering these experiences. For example, Mrs. F. reacted automatically and nearly off-handedly when she met “Elizabeth”. She neither abreacted nor was perplexed or baffled, since such a meeting was not out of the ordinary. Or, as Mrs. F. commented: “You get used to everything”. Her life-world was constantly double-fold for over 2 decades, as she was well aware. Consequently she did not expect this “other world” to cease to exist, although some of these (psychotic) “other-world-experiences” were annoying or even horrifying. On the contrary, it would have been truly astonishing if such apparitions would not have taken place anymore. In other words: she did not simply actively develop a delusional explanation for the changes in her world and experience, but developed a *delusional habituality* in addition to her common-sensical habituality. Her psychotic experiences were part of her familiar workspace, even though this familiarity did not imply a cozy feeling of being at home in her life-world. Accordingly persons with delusional convictions are typically able to provide reasons for their behaviour that are in accordance with these convictions [29] p. 108 a. 113 ff.

Obviously, one’s delusional insights and convictions provide only superficial knowledge regarding the improvement of one’s situation, as Mrs. F.’ experiences demonstrate quite clearly. Her delusional insights as well as her profound acquaintance with psychotic experiences (hallucinations, apparitions, delusional perceptions) did not really help her to change the givenness of the apparitions or other hallucinatory experiences and their delusional interpretation. This would, from a psychiatric vantage point, most probably support the regular use of a neuroleptic medication. She could, however, more easily cope with these hallucinations, being less startled and perplexed, and being able to distract herself actively from her otherwise constant awareness of “this other world”. These strategies were more or less habitualized and integrated in a highly structured daily routine, and supported her (relative) mental stability, helping her to feel at least partially “at home” in her life-world. Nonetheless, she recognized the limitations of her strategies and behavioural options. She located her impairments on the level of intentional action, but not on the level of free will: “It depends on how much strength you have, in order to come to term with things. That is important”. From a psychiatric perspective there is, however, a crucial and underlying impairment of free will, which is neither recognized nor reflectively attributed by Mrs. F. This refers primarily to the impossibility to de-select the delusional convictions and accept different and more reasonable, or common-sensical, explanations of her psychotic experiences, entailing different and possibly more effective strategies (i.e., including neuroleptic medication). Delusional convictions are obviously mixed blessings with respect to goals and strategies deriving from or depending on one’s delusional convictions, since they are usually neither achievable nor effective in a radical way. The “other world” was constantly the other side of her life-world, rendering her double-fold life-world both familiar and unhomelike at the same time.

### Limitations of lived autonomy in delusions

The missing effectivity to achieve goals with means founded on delusional convictions does not, contrary to what would be suspected, lead to a situation in which the deluded person reflectively re-frames these convictions (called *incorrigibility* in Jaspers’ sense). Similarly, neither the difference between one’s (delusional) convictions, and the knowledge, interpretations or convictions of one’s peer group, nor the acknowledgement that knowledge is, in principal, falsifiable, provoke a critical review. Instead, delusional convictions are pre-reflectively valued by the deluded person as “true” [4]. Furthermore, from the deluded person’s point of view, her delusional convictions are connected with her ideal of truth. In other words: the delusional convictions are not only pre-reflectively valued as true, they are also reflectively reconstructed as truth. This implies a profound intellectual incorrigibility of one’s delusional convictions. The incorrigibility is the most salient feature of missing effectivity in delusional disorders from a second- or third-person-perspective. Furthermore, it is, as already noted with reference to the case report, usually not even missed by the person with delusions, since it is an apparently not needed effectivity from her point of view (i.e., in the sense of the highest and most stable form of unintentional self-deception).

Profound incorrigibility on the level of a person’s existential convictions indicates a severe impairment of freedom of the will. Nonetheless, deluded persons experience their behaviour as self-determined when behaving according to their delusional convictions. I paraphrase here a famous description by Harry G. Frankfurt on (as we will soon discover, another form of) incorrigible convictions: The delusional person cannot help behaving in accordance with her delusional convictions. In this respect, she is not free. On the contrary, she is in the very nature of the case captivated by her delusionally interpreted and perceived objects and by her delusional convictions. Delusional convictions are, apparently, not a matter of choice (and, due to their ‘intensity’, they cannot even be perceived as a choice J.S.; [30], p. 135. Pointedly, Frankfurt’s actual description concerns the (pre-reflective) constraints *love* places upon our volitions. I simply changed words in the passage; the original states: “The person cannot help behaving in accordance with her love. In this respect, she is not free. On the contrary, she is in the very nature of the case captivated by her loved objects and by her love. […] Love is, apparently, not a matter of choice” [30], p. 135. Frankfurt concludes: “Our essential natures as individuals are constituted, accordingly, by what we cannot help caring about. The necessities of love, their relative order or intensity, define our volitional boundaries. They mark our volitional limits, and thus they delineate our shapes as persons” [30], p. 138. Surely we cannot simply compare love and delusions, even though such attempted comparisons could claim a long tradition in philosophical debates (e.g. as already started in Plato’s *Phaedrus*[31]). Following these passages we can, however, state the simple fact that delusional convictions are ‘dear’ to deluded people as if they were ‘in love’ with them (not implying that this involuntarily attachment must cause high feelings or render the ‘loved one’ a source of bliss). A person’s delusional convictions are intimately connected with this person’s everyday life; a deluded person behaves in accordance with her delusions because she simply cannot help herself with respect to being and staying convinced, even though her behaviour is in a specific manner ineffective. It is usually not very effective with respect to a) convincing others about the trueness of her convictions; b) achieving goals directly derived from her delusional convictions; and, especially, with respect to c) achieving these goals with strategies according to her delusional convictions. However, delusional convictions offer some kind of ‘epistemological’ rest by explaining the ongoing and disturbing changes in the world. This may be not much for us, but is usually very much for persons with delusions.

The important question regarding the freedom of the deluded person’s will is therefore similar to the same question with respect to people in love: Is the deluded person at least free to set out some goals which do not directly accord with her delusional convictions? If she can, she will most surely have a double-orientation to reality (Jaspers [6], p. 43 ff; Schwartz & Wiggins [8]). But if she can’t, she will most probably dwell within a profound ‘paranoid atmosphere’ which means that her delusional habituality and convictions completely dominate the manner in which she discloses her world to herself (see also Schlimme [4]). In the latter case, her ability to take another person’s perspective into account would be highly diminished if not lost. In other words: her competence to honour the goods of agency, not only for herself but also for others, would be specifically impaired due to her delusional customs and convictions. But virtuous and self-responsible agents are interested in treating others as agents themselves. This kind of capability is crucial for being truly autonomous, as John J Drummond argues: “The goods of agency (for Drummond these are at first: 1. making decision for oneself; 2. thinking well and truly about the situations in which one is called upon to act J.S.) are *necessary* conditions for the possibility of rightly ordering the goods for agents. These goods of agency must, therefore, be effectively willed in the virtuous pursuit of the goods for an agent. Moreover, since one cannot think or reason rightly by oneself, that is, since one must think *for* oneself but cannot rightly think *by* oneself, these goods of agency must be effectively willed for others as well as for oneself” [12], p. 45 f. The effective willing, or at least pre-reflective implication, of these goods of agency for others is a key differentiation between a primarily ‘subjectively autonomous’ behaviour, and a behaviour that can be attributed as autonomous (self-determined) also from another person’s perspective. We can therefore conclude that a deluded person’s experience of being free can be a correct and justifiable attribution, even if she is acting on her delusions (and in this way comparable to people in love). So, a behaviour can be called autonomous on a ‘framed’ level (level of freedom of intentional action), even though not being autonomous on a higher (‘framing’) level (i.e., level of freedom of the will), if, and only if, these goods of agency for others are truly respected.

The case of Mrs. F. demonstrated a number of such examples, indicating her ability to maintain a double-orientation to reality. She also reported experiences of lived autonomy on a reflective level that indicated a freedom of the will, but were typically not directly connected with her delusional convictions. To give an example for lived autonomy on the level of freedom of the will from Mrs. F’s life story, we can refer to her move to a major city two years ago as the most important decision of the last years. Her major arguments for the move, which she reported on enquiry were that a) several members of her immediate family lived there already for several years and had invited her to move (several times); b) no other relative remained in her former vicinity; and c) her best friend had died half a year before and that she had no other relevant social contacts in her locale. She summarized in one session: “All that shows me (referring to an overview of her actual life-situation debated before, J.S.) that my decision to have moved was as right as rain. That was obviously right”. As can be seen, the goods of agency of her loved ones and herself were respected in this decision at which she arrived after some months of intense consideration and discussions with her loved ones. Apparently delusional convictions or psychotic experiences did not directly influence this decision.

## Discussion

A person with delusions is not free to choose her delusional convictions. Her responsibility for her behaviour ends, in principal, on the level of these delusional convictions; comparable, at least in an analogous sense, with a person who is not responsible for being in love with someone else. In both cases, however, responsibility can extend to the behaviour which flows from these convictions. In this respect the person with delusions is, at least conceptually, not different from her fellow citizens who have fallen in love. A person with delusions is interpersonally embedded just like every other human being. Apparently, if a person is interested in being a moral agent, whether she is deluded or in love, she also needs to be interested in reflectively judging whether her valuings adequately correspond to her interests, and whether her interests are best served by the way she behaves. Consequently, a person with delusions who is a morally responsible agent, or wants to be, is interested in intersubjective verifications of her own valuings, behavioural options and interests and hence interested in the goods of agency for herself and others. To re-iterate, this entails that a person can be described as responsible for her behaviour on a ‘framed’ level (i.e., level of freedom of intentional action), even though she is not autonomous on a higher (‘framing’) level (i.e., level of freedom of the will), if, and only if, the goods of agency for others and herself are truly (at least not arbitrary) respected. In these cases the person with delusions could be called autonomous.

The inability to rectify one’s delusional convictions in reflection is, of course, not intentional. Various phenomenologically-informed psychiatrists have described this inability to reflectively reframe the delusional knowledge as a passively suffered inability [7,9,24,32-35]. Hence, being deluded is not a matter of choice, but of suffering. It is, however, usually supported by one’s perceptions. Or, to be more precise, it is supported by cognitively-perceived non-axiological properties; properties of the perceived objects’ or of one’s situation. If one’s delusional conviction could not be supported by one’s world in this manner, one would not stay convinced of these convictions, but would instead become aware that one’s (delusional) valuings and ideas are wrong, and would therefore sooner or later conclude that one was mistaken (for this argument, see especially Musalek [3]). Mrs. F. also received such worldly support in her perception. She repeatedly perceived translucent bodies, moving freely through walls, crowding her apartment and life-world, constraining her everyday-situations, commenting on her behaviour, and so forth, which she pre-reflectively valued and reflectively interpreted as ultimate proof for her (delusional) conviction of “the other world”.

From a phenomenological point of view, ongoing delusions and psychotic experiences supporting these delusions can, accordingly, be described as a specific style of habitualities. This specific style is, of course, a delusional style, and it further supports the delusional convictions. As Binswanger claimed, in delusional mental life, one’s cognition could (i.e., in severe psychotic states) both be reduced to the level of a mechanical registration (“*mechanische Registration*”) or impaired in the sense of a monotonous disposition of experiences (*“monotone Erfahrungsbereitschaft*”, [7], p. 469). Nonetheless, even if mental life of a person with delusions is not levelled to a mechanical registration or a monotonous disposition of experiences, as was the case with Mrs. F., her reflective activity is altered. On the one hand, she is unaware of her (delusional) convictions as self-deception; on the other hand, her imaginations are often primarily engaged with, and limited to the psychotic experiences themselves. This corresponds the finding that deluded people jump more often to conclusions in experimental settings ([36,37]; a thoughtful integration in [34,38]). Similarly, Hagen [26] argued that profound (existential) insecurity, in the sense of the primary delusional experience, makes it more necessary to achieve a delusional conclusion (see also Binswanger [7], p. 447 ff). This tendency of delusional habitualities to focus on the delusional topics indicates the limitedness of the freedom of the deluded person’s will.

To acknowledge that persons with delusions can be described as autonomous on the level of freedom of intentional action if, and only if, others are respected as (moral) agents, does not say that persons with delusions necessarily act autonomously. Acting on delusions can of course lead to immoral behaviour, because a person with delusions is unable to deselect her delusional convictions, and because she is profoundly and pre-reflectively influenced by her delusional habituality. Moreover, persons with delusions could also intentionally and responsibly act immorally or illegally, even though they are neither acting on delusions nor rendered unable to grant goods of agency for others. If, however, she discloses her life-world mainly, or solely in the manner of a delusional workspace, which could be called a ‘paranoid atmosphere’, she cannot take the perspective of others adequately into account [4]. Then the deluded person follows exclusively her “*Wahnlogik*” (logic of delusions), as Ludwig Binswanger argued with respect to August Strindberg’s concept of “fate’s logic”, [7], p. 536 ff. In this condition she is neither responsible for her delusional convictions, nor for her behaviour following these convictions. But this can be totally different if a person with delusions maintains a double-orientation to reality ([6], p. 101 “*doppelte Orientierung*”; see Binswanger [7], p. 536 ff; Sass [9]). Mrs. F., for example, managed to maintain such a double-orientation to reality for over 29 years. This double-orientation seems, however, to be frequent in chronic schizophrenia. Nonetheless, it is challenging and strenuous, because it is the person with delusions herself who primarily must integrate both, often controversial, realities [8]. Living in such a double-orientation to reality allows the person with delusions to select (delusional) strategies which enable her to (partially) achieve her (delusional) goals and, simultaneously, to respect relevant norms of her community and society. Therefore, it can also be addressed as an often eligible way of being in recovery for people with chronic schizophrenia [4,8,27,39,40].

To acknowledge that persons with delusions can behave self-determinedly, autonomously and responsibly does not deny (from a psychiatric point of view) that they are ill and could, or maybe even: should, be treated. They are, after all, most times at least partially “unhomelike” in their double-fold life-world, as were those of Mrs. F. This does not imply that deluded persons experience themselves as effective with respect to all aspects of their behaviour. They are usually unable to convince others about the trueness of their convictions. Furthermore, they are often ineffective in achieving goals and applying strategies directly deriving from, or relying upon, their delusional convictions. They are, however, typically able to achieve an explanation for the changes in their world. In this way, their freedom of intentional action is (in addition to symptom-related impairments of their free will) specifically altered. Persons with delusions are, as the case report of Mrs. F. most clearly demonstrated, able to experience themselves as free and self-determined in certain situations and actions; and they do in fact behave autonomously (and responsibly in a personal and legal sense) when taking into account the goods of agency in the situations in which they are called upon to act adequately. This is not denying the possibility of intentionally acting immorally, although the others’ goods of agency are accepted in principal and could be granted adequately. As has been argued above, such an adequateness is even possible if acting on one’s delusions. Although this need not be the case, and might seldom be, it seems to be especially possible if this person maintains a double-orientation to reality, implying that she discloses her life-world in two ‘parallel’ or ‘each other alternately eclipsing’ manners of familiar workspaces.

Therefore, our phenomenological investigation offers a possible answer to the question posed in this paper. Namely, yes, acting on delusions can indeed be autonomous, according to the person’s own experience of being free and self-determined, if the goods of agency are adequately respected not only for herself, but also for others. Persons with delusions are, in this respect, quite comparable to persons in love, who are also not free to choose their convictions, but can be held responsible for their behaviour flowing from their love. An important difference can be named in the simple fact that persons who have fallen in love tend to readily and deliberately grant others the goods of agency, especially those whom they love, while persons with delusions often have to struggle for maintaining goods of agency for others in their often double-fold familiar workspace. This seems to foster the difficulty to determine whether a person acting on delusions acted in fact autonomously, and was hence responsible for her behaviour, or not. Notably, not the manifest behaviour itself answers this question, but the adequateness of granted goods of agency for others in her familiar workspace, whether implied by virtues or integrated by explicit deliberation. Our phenomenological descriptions of lived autonomy in delusions, drawing on the classical and partial analogy between people in love and people with delusions, offer some clarifications regarding this most complex problem, but they remain descriptions nonetheless. The acknowledgement, however, that persons with delusions can behave autonomously, even if they act on their delusions, seem – at least to some extent – to be the grounds of a very traditional idea in psychiatry: that professionals should also try to be mediators between the different realities of their patients, even though this might sometimes not work.

## Endnote

^a^Mrs. F. is my patient treated regularly in the Outpatient Department of the Psychiatric University Hospital Charité at St Hedwig Hospital Berlin, Germany. She gave me informed consent to present her case and, specifically, to quote transcripts from some of our therapeutic sessions. “F.” is a pseudonym. Moreover, all patient information has been deindentified.

## Competing interest

The author declares that he has no competing interests.
